# Association between the neutrophil-to-lymphocyte ratio and risk of in-hospital heart failure and arrhythmia in patients with acute myocardial infarction

**DOI:** 10.3389/fcvm.2023.1275713

**Published:** 2023-10-20

**Authors:** Jia-li Zhang, Rui Yang, Yi Zhu, Yan Shao, Yuan Ji, Fang-fang Wang

**Affiliations:** ^1^ Department of Gastroenterology Centre, The Affiliated Changzhou Second People's Hospital of Nanjing Medical University, Changzhou, China; ^2^ Department of Clinical Pharmacy, Shandong Engineering and Technology Research Center for Pediatric Drug Development, Shandong Medicine and Health Key Laboratory of Clinical Pharmacy, The First Affiliated Hospital of Shandong First Medical University & Shandong Provincial Qianfoshan Hospital, Jinan, China; ^3^ Department of Cardiology, The Affiliated Changzhou Second People's Hospital of Nanjing Medical University, Changzhou, China

**Keywords:** NLR, AMI, STEMI, NSTEMI, in-hospital AHF, arrhythmia

## Abstract

**Background:**

This study was to probe into the relationship between the neutrophil-to-lymphocyte ratio (NLR) and both in-hospital and long-term heart failure risk in patients with acute myocardial infarction (AMI).

**Methods:**

990 patients with AMI, including 386 with non-ST-segment elevation myocardial infarction (NSTEMI) and 604 with segment elevation myocardial infarction (STEMI) were recruited between January 2019 and March 2022. The in-hospital acute heart failure (AHF) and arrhythmia events were recorded.

**Results:**

The NLR was significantly greater in the AHF group in STEMI and NSTEMI patients, with a higher frequency of arrhythmia in comparison to the non-AHF group. A high NLR was related to a high level of myocardial injury markers, accompanied with more AHF and arrhythmia events. Multivariate logistic regression analyses revealed that high NLR is independently linked with increased in-hospital AHF and arrhythmia risk. Receiver operating characteristic curve analyses revealed that the prognostic value of NLR for in-hospital AHF was 0.704 in STEMI patients and 0.766 in NSTEMI patients. However, during a median follow-up of 28 months with 32 heart failure patients, there was no significant difference between the low NLR group (*n* = 18) and the high NLR group (*n* = 14). Further analysis showed that the two groups did not significantly differ in the occurrence of heart failure within 12 months of discharge.

**Conclusion:**

Our results indicate that NLR is an independent risk factor of in-hospital AHF in AMI patients. However, NLR has no value in predicting long-term heart failure.

## Introduction

According to the 2021 China Cardiovascular Health and Disease Report, about 330 million patients have been suffered from cardiovascular and cerebrovascular diseases in China, including about 24 million patients with coronary heart disease and stroke. Acute myocardial infarction (AMI) has a high mortality rate, and although interventional treatment can markedly reduce the mortality rate, AMI prognosis remains relatively poor, and the incidence of acute heart failure (AHF) is high (1).

The neutrophil-to-lymphocyte ratio (NLR) is a well-established indicator in the study of cardiovascular disease prognostic models. A recent study found that the NLR is an important predictor of in-hospital death in AMI patients (2). In addition, NLR had predictive value in heart failure readmissions and prognosis in patients undergoing transcatheter aortic valve replacement (3). However, there is currently a lack of research on the potential use of NLR to predict the occurrence of in-hospital and long-term heart failure in patients with AMI. Although NLR does not directly reflect changes in cardiac inflammation and metabolic environment, it can be used to evaluate the prognosis of patients early on upon initial presentation to the emergency department. Furthermore, when combining NLR with classical markers of myocardial injury, the predictive value can be further improved. Moreover, due to the easy acquisition of NLR and the low cost to patients, a relatively complete cohort data can be obtained during follow-up.

During the course of clinical management of patients, a reciprocal causal relationship between heart failure and malignant arrhythmias was observed, in which both can be induced by the inflammatory environment. Additionally, major adverse cardiovascular events (MACE) in AMI patients following discharge represent a crucial research area that requires additional attention. It is found that NLR available at the time of admission could be applied for the evaluation of short-term outcomes in patients with cardiogenic shock as a complication of AMI, and higher NLR is an independent risk factor for elevated 30-day all-cause mortality and MACE (4). Besides, a study found that postoperative NLR (within 24 h) may be more effective in predicting the incidence of MACE in NSTEMI patients within one year after elective percutaneous coronary intervention (5). However, we found that most prognostic studies on NLR are confined to a duration of less than one year, and there is a lack of evidence for longer-term prognostic evaluations beyond this timeframe.

In the present study, the correlation between NLR and in-hospital AHF in patients with AMI was investigated. Moreover, we assessed the predictive utility of NLR for arrhythmia during hospitalization and MACE during the follow-up period.

## Patients and methods

### Participants

This study was carried out in accordance with the Declaration of Helsinki and was approved by the Ethics Committee of Clinical Investigation of The Affiliated Changzhou No.2 People's Hospital of Nanjing Medical University (KY314-01). Additionally, the study was registered in the China Clinical Trial Registration Center (ChiCTR2300067892). The clinical informed consent was collected from all subjects and/or their legal guardian(s), in ethics and consent section under declaration. We included a total of 1,058 AMI patients, and after exclusion criteria, 990 patients were ultimately included. A total of 990 patients with AMI were admitted in the study, including 386 with NSTEMI and 604 with STEMI, all of whom were admitted to the Affiliated Changzhou No. 2 People's Hospital of Nanjing Medical University between January 2019 and March 2022. The primary outcome measure was in-hospital AHF, and the secondary outcome measures were in-hospital arrhythmia and MACE.

The following inclusion criteria were used: (1) age between 18 and 80 years, (2) admitting diagnosis of AMI (STEMI and NSTEMI). Diagnostic criteria of STEMI and NSTEMI have been described in the previous study (6). Diagnostic criteria of STEMI: (1) chest pain history; (2) continuous elevation of ST segments (≥ 0.1 mV for more than 30 min, or V2 or V3 ≥ mV in two or more adjacent ECG leads), or new onset of left bundle branch block; (3) myocardial injury markers increased beyond the 99th percentile of the laboratory reference limit. Diagnostic criteria of NSTEMI: (1) a history of chest pain; (2) in leads with R wave dominant or R/S >1, a new horizontal or downwardly inclined ST segment depression (≥0.05 mV or T wave inversion ≥0.1 mV in two adjacent leads); (3) myocardial injury markers increased beyond the 99th percentile of the laboratory reference limit. The following exclusion criteria were used: (1) known history of heart failure or arrhythmia, (2) malignant tumor, (3) pregnancy, (4) severe liver dysfunction (decompensated liver cirrhosis, liver failure, etc), (5) severe hematological disorders (acute and chronic leukemia, lymphoma, etc), (6) history of coronary artery bypass grafting, (7) cardiogenic shock, (8) mechanical ventilation, and (9) mechanical circulatory support. The inclusion and follow-up process of this study population is consistent with previous studies (6, 7).

### Data retrieval and definitions

The Information related to demographics and clinical data during the patient's hospital stay was obtained from an electronic medical record system of hospital. The diagnosis of AHF was based on typical symptoms, signs, and laboratory examination, such as orthopnea, acute pulmonary edema, and B-type natriuretic peptide (BNP) levels, which was consistent with previous study (6). The definition of Arrhythmia is single or multiple episodes of atrial fibrillation, atrial flutter, ventricular fibrillation, or ventricular flutter, which was also consistent with previous study(6). The Gensini score was calculated to assess the severity of coronary artery disease, according to a previously described protocol (8).

### Follow-up

786 AMI patients recruited from January 2019 to August 2021 were followed up. Patients were interviewed one month after discharge and every three months afterward. General information, inpatient data, and medication situation were collected at each interview. Based on the information gathered during follow-up, outpatient visit was suggested if necessary. The study recorded MACE, which included all-cause mortality, heart failure, nonfatal MI, nonfatal stroke, and unplanned repeat revascularization (URR), and this information has been previously described in another study (7). The definition of heart failure during follow-up is consistent with that during hospitalization. Nonfatal MI was defined as new pathological Q waves in ≥2 contiguous electrocardiogram leads. Patients with acute ischemic cerebral vascular events were marked as stroke. Finally, URR was defined as any non-staged revascularization after Percutaneous Coronary Intervention (PCI).

### Statistical analyses

All continuous data have been normality tested. Normally distributed data were represented by the mean ± standard deviation, while skewed continuous data were represented by the median (interquartile range). Student's *t*-test or the Mann–Whitney *U*-test was performed to compare continuous variables between two groups, and the chi-squared test was used to compare categorical variables between two groups. Univariate and multivariate logistic regression analyses were conducted to assess the predictive value of the NLR for in-hospital AHF or arrhythmia risk. Receiver operating characteristic (ROC) curve analysis was performed to measure the cutoff value of the NLR for AHF during hospitalization. All tests were two-tailed, and *p* values <0.05 were considered significant. All statistical analyses were performed using IBM SPSS Statistics for Windows, version 22.0 (IBM Corp., Armonk, N.Y., USA).

## Results

The clinical characteristics of the 990 patients who participated in the study were presented in Table 1. The AHF events was recorded in 38 out of 604 STEMI patients, and in 51 out of 386 NSTEMI patients. Among the STEMI group, patients who had AHF were significantly older than those without AHF (64.9 ± 12.3 vs. 60.4 ± 14.2, *P* = 0.038). In both STEMI and NSTEMI patients, the proportion of concurrent arrhythmia events in the AHF patients was significantly higher than that in the non-AHF patients. Furthermore, The NLR was notably higher in the AHF group compared with the non-AHF group (10.5 ± 6.9 vs. 6.6 ± 5.6, *P* = 0.001).

**Table 1:** Baseline characteristics of the study population.

Characteristics	STEMI		*P*-value	NSTEMI		*P*-value
	Without AHF (*n* = 566)	With AHF (*n* = 38)		Without AHF (*n* = 335)	With AHF (*n* = 51)	
Age (years)	60.4 ± 14.2	64.9 ± 12.3	0.038	63.3 ± 13.1	65.6 ± 14.7	0.296
Sex, male, *n* (%)	459 (81.1)	31 (81.6)	0.941	241 (71.9)	33 (64.7)	0.289
BMI (kg/m^2^)	25.0 ± 4.1	25.1 ± 3.0	0.788	24.5 ± 4.2	24.2 ± 3.5	0.628
Smoking, *n* (%)	275 (50.9)	22 (59.5)	0.315	146 (46.8)	19 (38.8)	0.295
Hypertension, *n* (%)	342 (60.4)	28 (73.7)	0.104	218 (65.1)	37 (72.5)	0.294
Diabetes, *n* (%)	133 (23.5)	14 (36.8)	0.064	91 (27.2)	18 (35.3)	0.230
In-hospital arrhythmia, *n* (%)	28 (4.9)	17 (44.7)	<0.001	6 (1.8)	24 (47.1)	<0.001
Gensini score	49 (35–81)	53 (37–81)	0.690	29 (9–48)	60 (46–84)	< 0.001
Length of stay (days)	8.2 ± 2.9	8.0 ± 2.7	0.657	7.4 ± 2.6	8.5 ± 3.2	0.023
Biochemical test						
NEUT (*10^9^)	7.7 ± 3.1	9.9 ± 4.5	0.005	7.3 ± 3.2	9.3 ± 4.1	0.001
LYM (*10^9^)	1.5 ± 0.8	1.2 ± 0.6	0.012	1.5 ± 1.1	1.4 ± 0.8	0.536
NLR	6.6 ± 5.6	10.5 ± 6.9	0.001	6.3 ± 4.9	9.0 ± 7.2	0.012
UA (umol/L)	328 (272–392)	344 (287–418)	0.417	335 (277–410)	353 (309–418)	0.135
LDL-C (mmol/L)	2.74 ± 0.84	2.82 ± 1.00	0.671	2.54 ± 0.92	2.79 ± 1.37	0.209
HDL-C (mmol/L)	1.03 ± 0.28	1.09 ± 0.33	0.330	1.05 ± 0.29	0.97 ± 0.22	0.020
TC (mmol/L)	4.5 ± 1.2	4.6 ± 1.1	0.327	4.3 ± 1.2	4.5 ± 1.5	0.328
TG (mmol/L)	1.47 (1.04–2.10)	1.75 (1.12–2.14)	0.518	1.52 (1.13–2.16)	1.45 (1.17–1.90)	0.779
TyG	9.0 ± 0.7	9.1 ± 0.8	0.382	9.0 ± 0.7	9.1 ± 0.7	0.301
TG/HDL	2.0 ± 3.1	2.0 ± 1.9	0.974	2.1 ± 2.6	2.2 ± 2.9	0.807
CPK (U/L)	950 (312–2003)	1,132 (528–1,791)	0.517	154 (76–376)	236 (116–879)	0.010
CK-MB (U/L)	81 (35–167)	102 (47–144)	0.598	22 (16–42)	31 (19–78)	0.008
HBDH (U/L)	483 (276–824)	493 (349–718)	0.662	197 (151–324)	274 (188–444)	0.001
BNP (pg/ml)	291 (81–1,165)	394 (79–1,445)	0.703	456 (149–1,610)	913 (284–3,193)	0.006
HbA1c (%)	6.5 ± 1.5	6.9 ± 1.7	0.198	6.5 ± 1.5	6.8 ± 1.7	0.262
Ccr (ml/min)	69 ± 43	71 ± 33	0.775	63 ± 31	59 ± 29	0.466
Ultrasonic cardiogram						
LA (mm)	3.9 ± 0.5	3.9 ± 0.4	0.825	3.9 ± 0.5	4.0 ± 0.5	0.198
LV (mm)	5.3 ± 2.2	5.2 ± 0.6	0.571	5.2 ± 0.6	5.3 ± 0.6	0.142
EF (%)	50 ± 9	52 ± 8	0.159	55 ± 9	50 ± 13	0.020
Pharmacological intervention						
Double antiplatelet, *n* (%)	521 (92.4)	37 (97.4)	0.694	291 (87.4)	48 (94.1)	0.432
Anticoagulation, *n* (%)	19 (3.4)	0 (0)	0.493	11 (3.3)	2 (3.9)	0.829
β-block, *n* (%)	340 (60.1)	21 (55.3)	0.550	176 (52.5)	29 (56.9)	0.578
Statin, *n* (%)	559 (98.8)	37 (97.4)	0.382	331 (98.8)	51 (100)	0.497
ACEI/ARB, *n* (%)	176 (31.1)	12 (31.6)	0.956	106 (31.6)	14 (27.5)	0.538
ARNI, *n* (%)	150 (26.5)	10 (26.3)	0.944	107 (32.3)	8 (16.0)	0.019
SGLT2i, *n* (%)	131 (23.2)	5 (13.1)	0.151	93 (27.8)	5 (9.8)	0.006
MRA, *n* (%)	114 (20.1)	9 (23.7)	0.611	50 (14.9)	16 (31.4)	0.004

BMI, body mass index; AHF, acute heart failure; NLR, neutrophil-to-lymphocyte ratio; UA, uric acid; LDL-C, low-density lipoprotein cholesterol; HDL-C, high-density lipoprotein cholesterol; TC, total cholesterol; TG, triglycerides; BNP, brain natriuretic peptide; CPK, creatine phosphokinase; CK-MB, creatine kinase-MB; HBDH, hydroxybutyrate dehydrogenase; Ccr, creatinine clearance rate; LA, left atrium; LV, left ventricle; LVEF, left ventricular ejection factor; ARNI, angiotensin receptor-neprilysin inhibitor; SGLT2i, sodium-glucose cotransporter 2 inhibitor; MRA, mineralocorticoid receptor antagonist.

Values are shown as the means ± SD, median (interquartile range) or percentage.

In NSTEMI patients, the AHF group had a significantly higher Gensini score compared to the non-AHF group (60 (46–84) vs. 29 (9–48), *P* < 0.001). In NSTEMI patients, the AHF group had lower levels of HDL-C compared to the non-AHF group (0.97 ± 0.22 vs. 1.05 ± 0.29, *P* = 0.020), and higher levels of myocardial injury and heart failure markers, including creatine phosphokinase (CPK, 236 (116–879) vs. 154 (76–376), *P* = 0.010), creatine kinase-MB (CK-MB, 31 (19–78) vs. 22 (16–42), *P* = 0.008), hydroxybutyrate dehydrogenase (HBDH, 274 (188–444) vs. 197 (151–324), *P* = 0.001), and B-type natriuretic peptide (BNP, 913 (284–3,193) vs. 456 (149–1,610), *P* = 0.006). Furthermore, patients with AHF had lower Ejection Fraction (EF) values than those without AHF (50 ± 13 vs. 55 ± 9, *P* = 0.020). Additionally, we examined the medications administered to patients during hospitalization. The ratios of patients receiving angiotensin receptor-neprilysin inhibitor (ARNI) and sodium-glucose cotransporter 2 inhibitor (SGLT2i) therapies in the AHF group was markedly lower than that in the non-AHF group (8 (16.0) vs. 107 (32.3), *P* = 0.019; 5 (9.8) vs. 93 (27.8), *P* = 0.006, respectively), while an opposite fact was found for mineralocorticoid receptor antagonist (MRA) treatment (16 (31.4) vs. 50 (14.9), *P* = 0.004) (Table 1).

**Figure 1:**
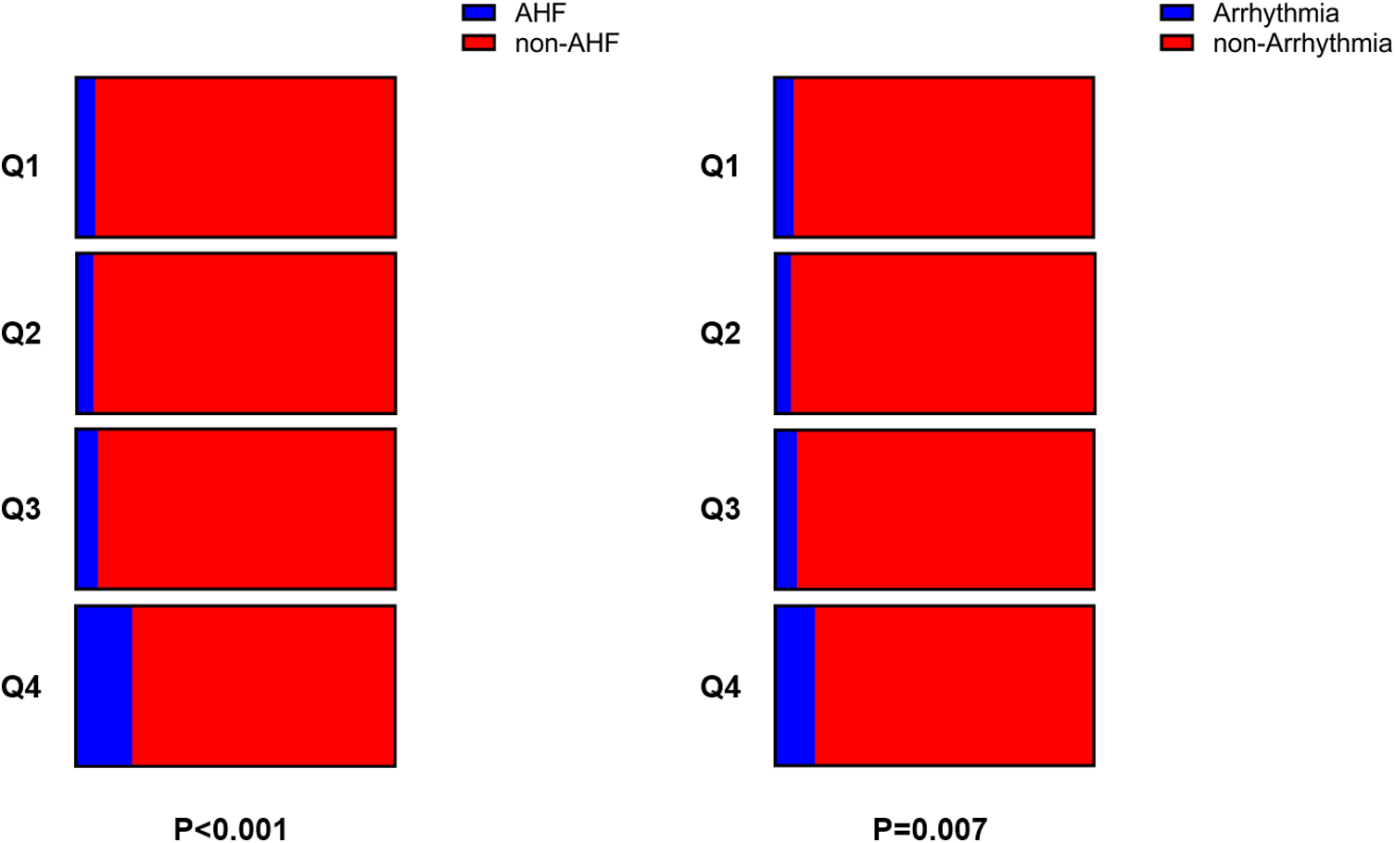
The proportion of patients with in-hospital AHF and arrhythmia in each of the 4 groups.

As BNP and myocardial injury indicators tests are used to assess the prognosis for in-hospital AMI patients, we grouped patients based on their NLR quartiles. The results indicated a significant increase in the levels of BNP, CPK, CK-MB and HBDH, with an increase in the NLR. Moreover, the proportion of patients with AHF and arrhythmia also increased significantly, particularly in the highest quartile (fourth quartile) group (Table 2 and Figure 1).

**Table 2:** The levels of myocardial injury markers and the occurrence of AHF and arrhythmia were correlated with NLR.

	NLR				*P* value
	Q1	Q2	Q3	Q4	
BNP (pg/ml)	285 (88–1,203)	362 (100–1,323)	419 (118–1,340)	477 (121–1,490)	0.003
CPK (U/L)	320 (105–1,067)	465 (132–1,178)	690 (171–1,672)	536 (158–1,540)	0.032
CK-MB (U/L)	38 (21–104)	44 (21–109)	60 (26–129)	55 (21–124)	0.015
HBDH (U/L)	298 (183–578)	332 (187–612)	395 (218–730)	357 (193–621)	0.308
AHF, *n* (%)	15 (6.0)	13 (5.3)	17 (6.9)	44 (17.7)	<0.001
Arrhythmia, *n* (%)	15 (6.0)	12 (4.9)	17 (6.9)	31 (12.5)	0.007

BNP, brain natriuretic peptide; CPK, creatine phosphokinase; CK-MB, creatine kinase-MB; HBDH, hydroxybutyrate dehydrogenase.

Both univariate and multivariate logistic regression analyses suggested that a high NLR was associated with an increased risk of in-hospital AHF in both STEMI (HR = 2.928, 95% CI: 1.393–6.158, *P* = 0.005) and NSTEMI patients (HR = 2.336, 95% CI: 1.236–4.416, *P* = 0.009) (Tables 3, 4). We also evaluated the potential of NLR in predicting arrhythmia occurrence during hospitalization. It was indicated that a high NLR was related with an increased risk of in-hospital arrhythmia in STEMI patients in both univariate and multivariate logistic regression analyses (HR = 2.133, 95% CI: 1.107–4.133, *P* = 0.024), but not in NSTEMI patients (Tables 5, 6).

**Table 3:** Univariate and multivariate logistic analyses for the in-hospital AHF risk in STEMI patients.

Parameters for AHF (STEMI)	Univariate analysis			Multivariate analysis		
	HR	95% CI	*P* value	HR	95% CI	*P* value
Age (years)	1.023	0.999–1.048	<0.001	1.025	1.000–1.050	0.051
Sex, male, *n* (%)	0.969	0.415–2.259	0.941			
Smoking, *n* (%)	1.413	0.718–2.783	0.317			
Hypertension, *n* (%)	1.834	0.874–3.849	0.109			
Diabetes, *n* (%)	1.899	0.955–3.776	0.067			
LDL-C (mmol/L) >3.4	0.991	0.443–2.218	0.983			
HDL-C (mmol/L) <1.04	0.907	0.466–1.765	0.774			
BNP (pg/ml) >400	1.081	0.560–2.085	0.816			
CPK (U/L) >200	1.331	0.507–3.498	0.562			
CK-MB (U/L) >20	1.084	0.372–3.154	0.883			
HBDH (U/L) >182	1.976	0.463–8.429	0.357			
NLR >median	2.860	1.364–5.998	0.005	2.928	1.393–6.158	0.005
β-block, *n* (%)	0.817	0.422–1.584	0.550			
ACEI/ARB, *n* (%)	1.020	0.503–2.068	0.956			
ARNI, *n* (%)	0.974	0.462–2.053	0.944			
SGLT2i, *n* (%)	0.501	0.192–1.309	0.158			
MRA, *n* (%)	1.222	0.563–2.655	0.612			

LDL-C, low-density lipoprotein cholesterol; HDL-C, high-density lipoprotein cholesterol; BNP, brain natriuretic peptide; CPK, creatine phosphokinase; CK-MB, creatine kinase-MB; HBDH, hydroxybutyrate dehydrogenase; ARNI, angiotensin receptor-neprilysin inhibitor; SGLT2i, sodium-glucose cotransporter 2 inhibitor; MRA, mineralocorticoid receptor antagonist.

**Table 4:** Univariate and multivariate logistic analyses for the in-hospital AHF risk in NSTEMI patients.

Parameters for AHF (NSTEMI)	Univariate analysis			Multivariate analysis		
	HR	95% CI	*P* value	HR	95% CI	*P* value
Age (years)	1.013	0.991–1.036	<0.001	1.003	0.980–1.027	0.795
Sex, male, *n* (%)	1.398	0.751–2.604	0.290			
Smoking, *n* (%)	0.720	0.389–1.334	0.296			
Hypertension, *n* (%)	1.418	0.747–2.730	0.295			
Diabetes, *n* (%)	1.463	0.785–2.726	0.231			
LDL-C (mmol/L) >3.4	1.190	0.563–2.513	0.649			
HDL-C (mmol/L) <1.04	1.526	0.820–2.840	0.182			
BNP (pg/mL) >400	1.690	0.900–3.172	0.102			
CPK (U/L) >200	1.439	0.797–2.597	0.227			
CK-MB (U/L) >20	1.681	0.886–3.190	0.112			
HBDH (U/L) >182	2.603	1.316–5.149	0.006	2.240	1.106–4.534	0.025
NLR >median	2.155	1.167–3.978	0.014	2.336	1.236–4.416	0.009
β-block, *n* (%)	1.183	0.653–2.144	0.579			
ACEI/ARB, *n* (%)	0.814	0.422–1.570	0.539			
ARNI, *n* (%)	0.399	0.181–0.879	0.156			
SGLT2i, *n* (%)	0.282	0.109–0.731	0.009	0.326	0.118–0.900	0.031
MRA, *n* (%)	0.597	0.337–1.041	0.005	0.872	0.342–2.143	0.007

LDL-C, low-density lipoprotein cholesterol; HDL-C, high-density lipoprotein cholesterol; BNP, brain natriuretic peptide; CPK, creatine phosphokinase; CK-MB, creatine kinase-MB; HBDH, hydroxybutyrate dehydrogenase; ARNI, angiotensin receptor-neprilysin inhibitor; SGLT2i, sodium-glucose cotransporter 2 inhibitor; MRA, mineralocorticoid receptor antagonist.

**Table 5:** Univariate and multivariate logistic analyses for the in-hospital arrhythmia risk in STEMI patients.

Parameters for arrhythmia (STEMI)	Univariate analysis			Multivariate analysis		
	HR	95% CI	*P* value	HR	95% CI	*P* value
Age (years)	1.034	1.010–1.057	0.005	1.024	0.998–1.051	0.068
Sex, male, *n* (%)	2.323	1.205–4.480	0.012	1.103	0.408–2.979	0.847
Smoking, *n* (%)	0.630	0.338–1.177	0.147			
Hypertension, *n* (%)	1.437	0.748–2.762	0.277			
Diabetes, *n* (%)	2.222	1.186–4.164	0.013	1.993	1.047–3.791	0.036
LDL-C (mmol/L) >3.4	1.568	0.797–3.085	0.192			
HDL-C (mmol/L) <1.04	0.742	0.403–1.365	0.337			
BNP (pg/ml) >400	2.055	1.091–3.868	0.026	1.629	0.826–3.210	0.159
CPK (U/L) >200	1.083	0.469–2.500	0.851			
CK-MB (U/L) >20	1.325	0.460–3.822	0.602			
HBDH (U/L) >182	1.528	0.458–5.092	1.528			
NLR >median	2.022	1.064–3.840	0.032	2.133	1.107–4.133	0.024
β-block, *n* (%)	0.826	0.448–1.523	0.540			
ACEI/ARB, *n* (%)	0.696	0.345–1.406	0.313			
ARNI, *n* (%)	1.119	0.572–2.191	0.742			
SGLT2i, *n* (%)	0.846	0.397–1.804	0.666			
MRA, *n* (%)	0.829	0.376–1.829	0.643			

LDL-C, low-density lipoprotein cholesterol; HDL-C, high-density lipoprotein cholesterol; BNP, brain natriuretic peptide; CPK, creatine phosphokinase; CK-MB, creatine kinase-MB; HBDH, hydroxybutyrate dehydrogenase; ARNI, angiotensin receptor-neprilysin inhibitor; SGLT2i, sodium-glucose cotransporter 2 inhibitor; MRA, mineralocorticoid receptor antagonist.

**Table 6:** Univariate and multivariate logistic analyses for the in-hospital arrhythmia risk in NSTEMI patients.

Parameters for arrhythmia (NSTEMI)	Univariate analysis			Multivariate analysis		
	HR	95% CI	*P* value	HR	95% CI	*P* value
Age (years)	1.010	0.982–1.039	0.499			
Sex, male, *n* (%)	1.245	0.563–2.752	0.588			
Smoking, *n* (%)	0.706	0.324–1.542	0.383			
Hypertension, *n* (%)	1.756	0.733–4.207	0.206			
Diabetes, *n* (%)	1.780	0.140–1.780	0.140			
LDL-C (mmol/L) >3.4	0.716	0.241–2.122	0.546			
HDL-C (mmol/L) <1.04	2.534	1.060–6.058	0.037	2.388	0.989–5.766	0.053
BNP (pg/ml) >400	1.490	0.678–3.276	0.321			
CPK (U/L) >200	0.926	0.437–1.964	0.842			
CK-MB (U/L) >20	0.984	0.460–2.105	0.966			
HBDH (U/L) >182	1.739	0.774–3.903	0.180			
NLR >median	1.660	0.777–3.547	0.191			
β-block, *n* (%)	0.868	0.412–1.830	0.711			
ACEI/ARB, *n* (%)	1.114	0.505–2.458	0.790			
ARNI, *n* (%)	0.458	0.170–1.233	0.017	0.510	0.167–1.559	0.011
SGLT2i, *n* (%)	0.715	0.283–1.803	0.477			
MRA, *n* (%)	1.527	0.626–3.722	0.352			

LDL-C, low-density lipoprotein cholesterol; HDL-C, high-density lipoprotein cholesterol; BNP, brain natriuretic peptide; CPK, creatine phosphokinase; CK-MB, creatine kinase-MB; HBDH, hydroxybutyrate dehydrogenase; ARNI, angiotensin receptor-neprilysin inhibitor; SGLT2i, sodium-glucose cotransporter 2 inhibitor; MRA, mineralocorticoid receptor antagonist.

**Figure 2:**
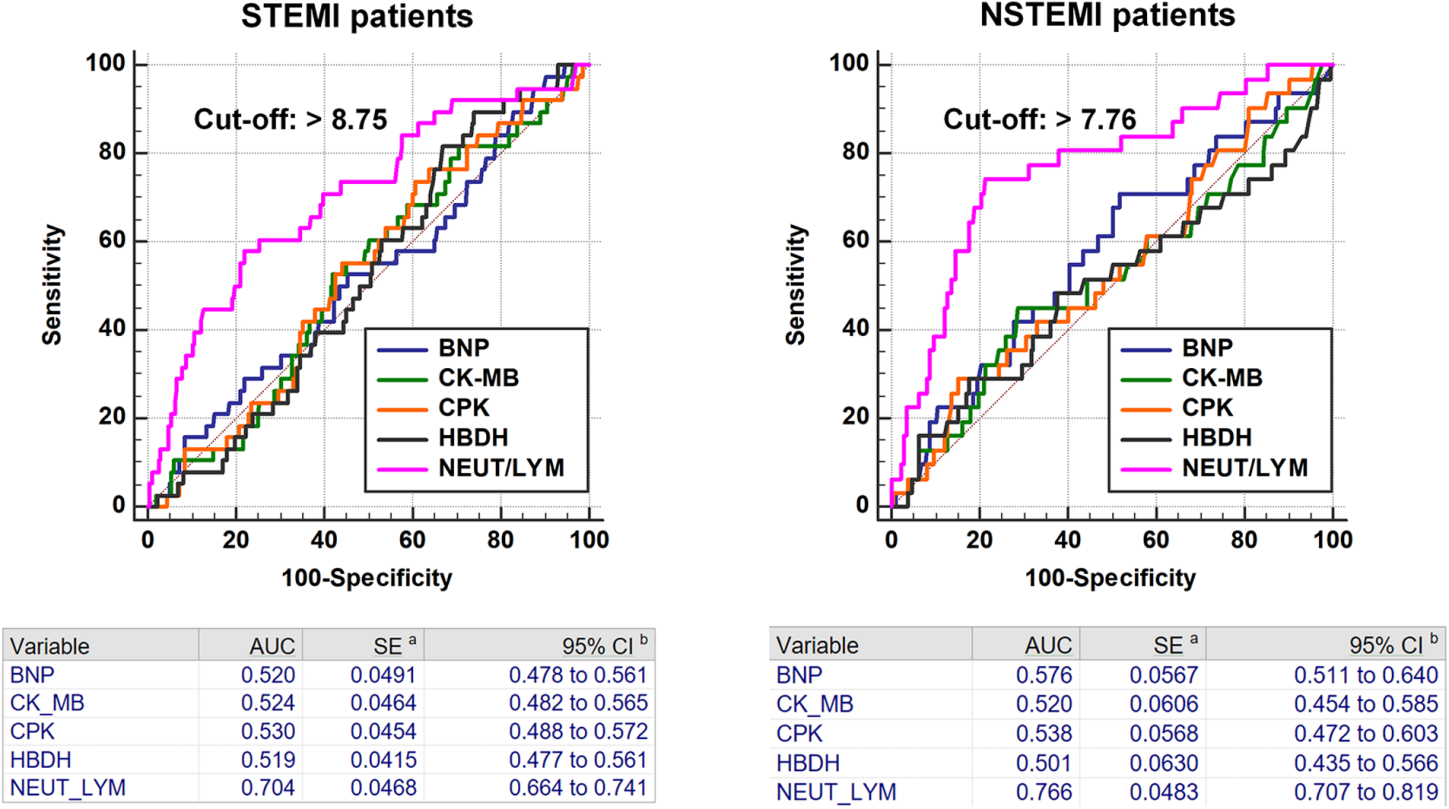
ROC curve analyses of BNP, CK-MB, CPK, HBDH and NLR for in-hospital AHF.

The ROC curve analyses indicated that NLR had areas under the curve of 0.704 (95% CI: 0.664–0.741, *P* = 0.002, cut-off value: >8.75) in STEMI patients and 0.766 (95% CI: 0.707–0.819, *P* < 0.001, cut-off value: >7.76) in NSTEMI patients for predicting in-hospital AHF occurrence (Figure 2).

Finally, we investigated the association of the NLR and long-term AHF occurrence. As presented in Table 7, the prevalence of overall death, heart failure, non-fatal MI, non-fatal stroke and URR had no significant difference between the low and high NLR groups. Since NLR is a short-term inflammation indicator, we then analyzed the correlation between the NLR and AHF occurrence within 12 months of discharge. Similarly, there was no significant difference observed in overall mortality, heart failure, non-fatal MI, non-fatal stroke and URR between the low and high NLR groups during the 12-month period following discharge (Supplementary Table S1).

**Table 7:** Adverse cardiovascular events during follow-up.

Adverse Cardiovascular events	NLR <median	NLR >or = median	*P* value
MACE, *n* (%)	64 (12.4)	66 (11.8)	0.799
Overall death, *n* (%)	4 (1.0)	6 (1.0)	0.968
Heart failure, *n* (%)	33 (4.7)	37 (3.5)	0.722
Non-fatal MI, *n* (%)	38 (5.2)	43 (6.0)	0.600
Non-fatal stroke, *n* (%)	8 (2.1)	7 (1.0)	0.825
URR, *n* (%)	55 (9.0)	54 (10.3)	0.751

MACE, major adverse cardiovascular events; MI, myocardial infarction; URR, unplanned repeat revascularization.

The MACE was defined as the composite of overall death, heart failure, non-fatal MI, non-fatal stroke, and URR.

## Discussion

The study was to assess the association between NLR and in-hospital heart failure in AMI patients. The findings revealed that AHF patients had a significantly higher NLR with a higher incidence of arrhythmia compared to the non-AHF group. In addition, elevated NLR was independently associated with increased risk of in-hospital AHF and arrhythmia. NLR was predictive of in-hospital AHF in AMI patients, however, NLR was not a predictor of long-term heart failure in AMI patients.

Following myocardial infarction, necrosis of the myocardium can lead to an inflammatory storm that can further disrupt the homeostasis of the myocardial environment, potentially resulting in adverse cardiovascular events (9). In this context, the neutrophil-to-lymphocyte ratio is a readily available, inexpensive, and credible indicator of systemic inflammation, and thus has been evaluated in our study as a potential biomarker for predicting in-hospital and long-term cardiovascular outcomes.

Due to the standardization of clinical diagnosis and treatment, patients with suspected AMI are now subjected to laboratory investigations, including blood routine tests, myocardial enzyme profiling, and coagulation tests upon their initial admission to the emergency department. Furthermore, NLR has been confirmed to possess a good predictive value in evaluating the degree of sepsis and local inflammation (10, 11). Inflammatory markers, including NLR, are also influenced by the patient's hyperglycaemic state and that it also has an impact on long-term prognosis (12, 13). In this setting, NLR can be obtained at an early stage as an inflammatory indicator.

Inflammatory storms are triggered by various factors such as trauma, shock, infection, and injury (14). Firstly, mononuclear macrophages activate inflammatory cells by presenting antigens through natural immunity. These inflammatory cells then release cytokines, which enter the blood circulation and act on different cells including, white blood cells, red blood cells, platelets, and vascular endothelial cells (15, 16). This action leads to the production of platelet aggregation factor, prostaglandin, peroxidase synthase, leukotriene, nitric oxide, and other cytokines, which can increase the concentration of C-reactive protein, *α*2-macroglobulin, and fibrinogen while decreasing the concentration of albumin and transferrin (17, 18). Ultimately, these effects lead to high discharge and low resistance of the entire heart circulation, eventually resulting in heart failure (19, 20). Our study revealed a significant increase in NLR in the AHF group among STEMI and NSTEMI patients. Conversely, there was no significant difference in other clinical indicators between the AHF and non-AHF groups in STEMI patients. It is noteworthy that STEMI patients typically undergo emergency PCI surgery upon admission, which can offer substantial protection to the heart from injury. However, significant differences in many traditional indicators were observed between the AHF and non-AHF groups in the NSTEMI group. This could be attributed to the fact that NSTEMI patients usually have more diffuse coronary artery lesions, and interventional treatment is usually elective surgery, leading to more severe inflammation and myocardial injury in patients with AHF. Multivariate logistic regression and ROC analyses suggested that a high NLR is associated with an increased risk of in-hospital AHF. Based on the data obtained from this study, it can be concluded that a high NLR can predict the occurrence of in-hospital AHF to a certain extent.

In this study, we analyzed the predictive value of NLR in arrhythmia. Logistic regression analysis showed that a high NLR is an independent predictor of arrhythmia in STEMI patients during hospitalization. We also observed that the AHF group had a high proportion of patients who have arrhythmia in STEMI and NSTEMI patients. Arrhythmia can lead to hemodynamic disorders and exacerbate cardiac workload. In addition, the occurrence of AHF activates the sympathetic nervous system *in vivo*, which triggers abnormal pacemakers in the heart cavity (21). Therefore, a reciprocal cause-and-effect relationship between NLR and arrhythmia has been reported in several studies. Elevated NLR significantly increases the risk of left atrial thrombosis in non-valvular atrial fibrillation patients, where NLR is an independent risk factor. Moreover, NLR is an independent predictor of long-term outcomes in patients with atrial fibrillation (AF) (22)., while a high NLR is associated with an increased risk of new-onset AF (23). Noteworthy, the small sample size in this study may have contributed to the lack of statistical significance in the predictive value of NLR in patients with NSTEMI.

The primary purpose of this study was to investigate the effect of SGLT2i on the risk of cardiovascular and cerebrovascular adverse events in patients with AMI. Therefore, we extracted the health and medical information of patients after discharge. The protective role of SGLT2i on in-hospital outcome in AMI patients was consistent with the previous studies (24–26). In this study, there was no statistically association between the NLR ratio and the incidence of long-term heart failure. This is likely due to the fact that the NLR data were obtained from patients admitted to the emergency department and is indicative of short-term inflammation levels. Clinical cohort studies examining the association between NLR and long-term outcomes in AMI patients may provide valuable insights into the management of these patients.

## Limitations

This single-center, retrospective study, was limited by a potential bias caused by confounding factors. However, univariate and multivariate logistic regression analyses were employed to mitigate the interference of such confounding factors. Besides, the results may not be generalizable to populations of different ethnic backgrounds and over 80 years of age. The small sample size is also a limitation.

## Conclusion

Our study found that a high NLR was independently related with an increased risk of in-hospital AHF and arrhythmia in patients with acute myocardial infarction. Furthermore, NLR was found to be a predictive marker for in-hospital AHF in AMI patients. However, a high NLR could not predict long-term heart failure in AMI patients. These findings provide further evidence for the relationship between NLR and the prognosis of AMI patients, which is expected to play a role in the clinical management of AMI patients.

## Data Availability

The raw data supporting the conclusions of this article will be made available by the authors, without undue reservation.
