# Supplementary material for: Association between the neutrophil-to-lymphocyte ratio and risk of in-hospital heart failure and arrhythmia in patients with acute myocardial infarction
Source: Front Cardiovasc Med. 2023 Oct 20;10:1275713. doi: 10.3389/fcvm.2023.1275713 (PMC10623153; doi:10.3389/fcvm.2023.1275713)
Supplement: Supplementary file 1 [file Table1.docx]

**Supplementary Table 1 Adverse cardiovascular events within 12 months of discharge.**

| **Adverse Cardiovascular events (12 months)** | **NLR < median** | **NLR > or = median** | **P value** |
| --- | --- | --- | --- |
|  |  |  |  |
| MACE, n (%) | 24 (6.6) | 25 (6.6) | 0.984 |
| Overall death, n (%) | 3 (0.8) | 3 (0.8) | 0.640 |
| Heart failure, n (%) | 7 (1.9) | 8 (2.1) | 0.848 |
| Non-fatal MI, n (%) | 9 (2.5) | 15 (4.0) | 0.247 |
| Non-fatal stroke, n (%) | 6 (1.7) | 1 (0.3) | 0.056 |
| URR, n (%) | 14 (3.9) | 22 (5.9) | 0.208 |

The MACE was defined as the composite of overall death, heart failure, non-fatal MI, non-fatal stroke, and URR.

MACE: major adverse cardiovascular events; MI: myocardial infarction; URR: unplanned repeat revascularization.
